# The relationship between areas of life satisfaction, personality, and overall life satisfaction: An integrated account

**DOI:** 10.3389/fpsyg.2022.894610

**Published:** 2022-09-21

**Authors:** Antonio Malvaso, Weixi Kang

**Affiliations:** ^1^Neuroimaging Research Unit, Division of Neuroscience, IRCCS San Raffaele Scientific Institute, Milan, Italy; ^2^Neurology Unit, IRCCS San Raffaele Scientific Institute, Milan, Italy; ^3^School of Medicine and Surgery, Vita-Salute San Raffaele University, Milan, Italy; ^4^Computational, Cognitive and Clinical Neuroimaging Laboratory, Division of Brain Sciences, Imperial College London, London, United Kingdom

**Keywords:** personality, Big Five, life satisfaction, job satisfaction, health satisfaction

## Abstract

A substantial amount of research has been conducted using a variety of methodological approaches to determine what influences life satisfaction. The bottom-up theory considers overall life satisfaction as a function of various areas of life satisfaction, whereas the top-down theory considers the areas of life satisfaction as a function of dispositional factors such as personality. We examined these models in a large-scale United Kingdom survey. Consistent with other studies, we found that both the bottom-up and top-down models of life satisfaction are supported in the United Kingdom by demonstrating that demographics, areas of life satsifaction, and personality traits can explain a significant portion of variances in overall areas of life satisfaction. We propose that future studies in life satisfaction research should consider the integrated account of life satisfaction rather than a unitary bottom-up or top-down perspective.

## Introduction

The term “life satisfaction” relates to how much a person likes their life (Diener et al., 1998). During the last few decades, many studies investigated that can predict life satisfaction. Life satisfaction is a cognitive and global assessment of one’s overall quality of life. In particular, there are numerous variables that influence life satisfaction, including sociodemographic factors like health, job, household, family, age, gender, psychological characteristics, lifestyle, leisure activity involvement, and leisure enjoyment (Rojas, 2006; Agyar, 2013; Magee et al., 2013; Moksnes and Espnes, 2013; Loewe et al., 2014; Newman et al., 2014; Kuykendall et al., 2015).

Two theories in life satisfaction research have been discussed intensely: the bottom-up and top-down theory (Diener, 1984; Headey et al., 1993; Erdogan et al., 2012; Loewe et al., 2014). The bottom-up theory looks at overall satisfaction as a function of several aspects of life satisfaction (Erdogan et al., 2012). Individuals’ responses to questions on their life satisfaction, according to the bottom-up perspective, are a complex function of satisfaction with many life domains. Life satisfaction is not a straightforward average of domain satisfaction as people assess each domain differently. While some people consider leisure to be the most essential aspect of their lives, others prioritize job or health (Diener et al., 2003; Newman et al., 2014). Satisfaction with domains that are consistent with one’s values has been demonstrated to be more essential for one’s overall satisfaction (Oishi et al., 1999). Those who place a high value on success and those who place a high value on relationships, for example, will place a different emphasis on job and family satisfaction in their life satisfaction assessments. However, when the influences of personality and nonwork satisfaction are taken into account, job satisfaction does not predict life satisfaction (e.g., Rode, 2004). Furthermore, discontent in one domain frequently leads to a reevaluation of that domain’s relevance (Wu et al., 2009). For someone suffering from health issues, this could include a greater emphasis on family contentment.

The top-down theory views overall life satisfaction or specific areas of life satisfaction as a result of personality and other stable characteristics (Diener, 1984; Loewe et al., 2014). In this sense, life satisfaction is determined by personality disposition (which manifests in relatively stable cognitive and affective qualities, resulting in an individual displaying stable behavior—for a summary, see Montag and Panksepp, 2017). Steel et al. (2008) conducted a meta-analysis of 249 studies and found that the Big Five explained 18% of total variances in life satisfaction. Neuroticism is the most related (*β* = −0.30) and the rest less related (Extraversion, *β* = 0.17; Conscientiousness, *β* = 0.07; Openness, *β* = −0.04; Agreeableness, *β* = 0.03). A meta-analysis of 137 studies conducted by DeNeve and Cooper (1998) found that characteristics dealing with emotional expression (such as emotional stability) and traits relating to how life events are understood (such as defensiveness) were the strongest correlations of life satisfaction and overall life satisfaction. Another meta-analysis done by Heller et al. (2004) showed that employment and marital satisfaction mediated the effects of personality on life satisfaction. In other words, top-down factors may influence life satisfaction through shaping views of life domains.

According to Erdogan et al. (2012), top-down effects (e.g., personality) might influence the perception of many aspects of life satisfaction, hence affecting overall life satisfaction. They also advised that personality be used as a distal predictor rather than a control variable in models of life satisfaction (Erdogan et al., 2012). However, in models of life satisfaction, characteristics such as age, gender, and education should be adjusted for (Diener, 1984; Gutiérrez et al., 2005). Several research suggested that these variables may influence the relationship between personality and/or specific life satisfaction characteristics and overall satisfaction, although findings are not always consistent (Diener, 1984; Magee et al., 2013; Moksnes and Espnes, 2013; Ulloa et al., 2013). Furthermore, there has been research into the relationship between personality and overall life satisfaction, the results have been mixed (Costa and McCrae, 1980; DeNeve and Cooper, 1998; Schimmack et al., 2004; Asthana, 2011; Baudin et al., 2011; Gale et al., 2013; Hosseinkhanzadeh and Taher, 2013; Kjell et al., 2013). Personality psychologists generally believe that five major dimensions may appropriately organize a large range of possible personality characteristics. Extraversion, Agreeableness, Conscientiousness, Neuroticism, and Openness are the five “super traits” that comprise the Big Five (John and Srivastava, 1999; Rammstedt and John, 2007; Asthana, 2011). Neuroticism and Extraversion are often the strongest predictors of life satisfaction in research investigating the link between personality traits and life satisfaction (Diener and Lucas, 1999; Lachmann et al., 2017). Lachmann et al. (2017) used demographic variables, personality variables, and areas of life satisfaction variables to build a stepwise multiple regression model to predict overall life satisfaction. They discovered that demographic and personality variables could only explain 0.1%–1.8% of the variance in overall life satisfaction (Lachmann et al., 2017). However, when personality variables and various areas of life satisfaction variables were entered in a hierarchical regression model in separate blocks, the explained variance (*R*^2^) of all personality variables did increase to a maximum of *R*^2^ = 0.098.

Additionally, compensation, spillover, and segmentation effects are mechanisms that help explain the complex link between areas of life satisfaction and overall life satisfaction (Rojas, 2006). A compensation effect proposes a negative relationship between areas of life satisfaction and/or overall life satisfaction, whereas a spillover effect suggests a positive relationship (Erdogan et al., 2012). The phrase segmentation is used when modifications in one area have no effect on other areas and/or total life satisfaction (Erdogan et al., 2012). Rojas (2006) discovered more details on the nature of the relationships between different aspects of life satisfaction and overall life satisfaction. In this case, dispositional elements would determine how satisfied a person is, providing an inference on their level of contentment based on their personality structure. For example, Extraversion or Neuroticism could provide researchers with insight about how satisfied a person should be (Diener, 1984; DeNeve and Cooper, 1998; Heller et al., 2004). In fact, although there are mostly positive relationships between Extraversion and life satisfaction, the link between Neuroticism and life satisfaction is almost always negative (Diener, 1984). Several meta-analyses have also confirmed these kinds of connections (DeNeve and Cooper, 1998; Heller et al., 2004; Steel et al., 2008).

Taken together, these findings show that neither the bottom-up nor a top-down theory can adequately explain life satisfaction on their own. Instead, an integrated view that incorporates both models might be the most effective. Despite the fact that both theories are sometimes represented as opposing models (Loewe et al., 2014), there have been various attempts to combine the bottom-up and top-down theories in a single integrated model (Feist et al., 1995; Heller et al., 2004; Newman et al., 2014; Busseri, 2015; Lachmann et al., 2017). In particular, they found that both personality and areas of life satisfaction explained some portion of total variances in overall life satisfaction.

Overall satisfaction can be defined in general terms to some extent, but it must be understood in the context of each culture. Furthermore, Tov and Diener (2009) demonstrated that while there are pancultural experiences of general satisfaction that can be compared across cultures, there are also culture-specific patterns that distinguish cultures in their satisfaction experiences. Moreover, some research studies have investigated cross-cultural differences in life satisfaction (Diener and Suh, 2000; Park et al., 2004; Diener and Diener, 2009). In particular, various cultures interpreted life satisfaction differently: for example, individuals living in an individualistic society are more concerned with their personal aims, interests, and feelings than with the well-being of a community (e.g., friends or family). On the other hand, harmonious connections with other people are valued more than personal aims in more collectivistic civilizations. In collectivistic societies, family satisfaction may be rated higher than in individualistic cultures (Park et al., 2004). Furthermore, disparities in life satisfaction levels between nations have been discovered Loewe et al. (2014). People living in individualistic cultures reported higher levels of life satisfaction than those in collectivistic cultures. As suggested by Loewe et al. (2014), this could be explained by the fact that in individualistic cultures, the personal, goal-oriented perspective contributes to more self-referred attribution of failure and success, perhaps leading to better overall life satisfaction when compared to persons in collectivistic cultures. However, few studies have used such a large sample from the United Kingdom to investigate the top-down and bottom-up theories. Hence, we investigated personality and areas of life satisfaction in the United Kingdom.

The aim of our study is to investigate if bottom-up or top-down theories, or an integrated account of life satisfaction are supported in the United Kingdom. If bottom-up theories are supported, then we would expect that demographics and different areas of satisfaction can significantly predict overall satisfaction. On the other hand, if dimensions of personality are significant predictors of overall satisfaction, then the top-down theories would be valid. We would expect to see both personality and areas of life satisfaction explain some portion of the overall life satisfaction variances if an integrated account of life satisfaction is supported.

## Materials and methods

### Data

We used data from the British Household Panel Study (BHPS; University of Essex, 2018), which has been collecting data on representative samples of individual households in the United Kingdom since 1991. Wave 15 data are collected from September 2005 to May 2006. Personality traits including Neuroticism (*α* = 0.68), Openness (*α* = 0.66), Agreeableness (*α* = 0.54), Conscientiousness (*α* = 0.53), Extraversion (*α* = 0.59) were measured using the 15-item version of the Big Five Inventory with a Likert scale ranging from 1 (“disagree strongly”) to 5 (“agree strongly”). Participants responded to questions that asked about their overall satisfaction and satisfaction with health, income of household, house/flat, spouse/partner, job, social life, amount of leisure time, and use of leisure time with a Liker scale ranging from 1 (“Not satisfied at all”) to 7 (“Completely satisfied”). According to Lucas and Donnelan (2007), the reliability of this single-item measurement of overall life satisfaction is at least 0.67. Participants also completed questionnaires asking them about their demographics including: age, sex, marital status, highest educational qualification, political party supported, and employment status. We excluded participants who were younger than 16 or who are older than 99 and who had missing fields in variables that we are interested in. Hence, 5,928 data points survived from the original 15,617 participants in the study. Descriptive statistics for these variables is found in Table 1.

**Table 1:** Descriptive statistics for continuous and categorical variables.

**Variable name**	**Mean**	**SD**	**Medians**	**Ranges**	**Skewness**	**Kurtosis**
Age	41.37	12.29	41.00	16.00–84.00	0.19	2.49
Satisfaction with health	5.02	1.40	5.00	1.00–7.00	−0.79	3.23
Satisfaction with income of household	4.58	1.45	5.00	1.00–7.00	−0.49	2.76
Satisfaction with house/flat	5.33	1.32	6.00	1.00–7.00	−0.95	3.80
Satisfaction with spouse/partner	6.16	1.20	7.00	1.00–7.00	−1.86	6.72
Satisfaction with job	5.05	1.43	5.00	1.00–7.00	−0.84	3.46
Satisfaction with social life	4.82	1.35	5.00	1.00–7.00	−0.47	2.93
Satisfaction with amount of leisure time	4.29	1.50	4.00	1.00–7.00	−0.19	2.45
Satisfaction with use of leisure time	4.62	1.42	5.00	1.00–7.00	−0.34	2.65
Overall life satisfaction	5.26	1.09	5.00	1.00–7.00	−0.82	4.02
Neuroticism	3.61	1.24	3.67	1.00–7.00	0.21	2.80
Openness	5.43	0.95	5.67	1.00–7.00	−0.59	3.33
Agreeableness	4.56	1.13	4.67	1.00–7.00	−0.07	2.73
Conscientiousness	5.44	0.98	5.67	1.00–7.00	−0.43	2.85
Extraversion	4.58	1.11	4.67	1.00–7.00	−0.25	3.10
**Variable name**	**Value**		**Count (n)**		**Percent (%)**	
Present legal marital status	Married		8,115		51.96	
	Separated		327		2.09	
	Divorced		1,248		7.99	
	Widowed		1,192		7.63	
	Never married		4,727		30.27	
Highest educational qualification	Higher degree		425		2.7	
	First degree		1,597		10.2	
	Teaching QF		339		2.2	
	Other higher QF		3,432		22.0	
	Nursing QF		161		1.0	
	GCE A levels		1811		11.6	
	GCE O levels or equi		2,517		16.1	
	Commercial QF, No O		331		2.1	
	CSE Grade 2–5, Scot G		421		2.7	
	Apprenticeship		257		1.6	
	Other QF		102		0.7	
	No QF		2,787		17.8	
	Still at school No Q		138		0.9	
Political party supported	Conservative		2,372		15.2	
	Labor		3,964		25.4	
	Lib Dem/Lib/SDP		1,606		10.3	
	Scot Nat		396		2.5	
	Plaid Cymru		206		1.3	
	Green party		179		1.1	
	Other party		142		0.9	
	Other answer		54		0.3	
	None		2,213		14.2	
	Cannot vote		287		1.8	
	Ulster Unionist		480		3.1	
	SDLP		457		2.9	
	Alliance party		119		0.8	
	Democratice Unionist		504		3.2	
	Sinn Fein		233		1.5	
	Other party		42		0.3		**Variable name**	**Value**	**Count (n)**	**Percent (%)**
Sex	Male		7,120		45.6	
	Female		8,497		54.4	
Employment status	Self employed		1,030		6.60	
	In paid employ		7,318		46.86	
	Unemployed		486		3.11	
	Retired		3,074		19.68	
	Maternity leave		94		0.60	
	Family care		953		6.10	
	FT studt, school		877		5.62	
	LT sick, disabled		656		4.20	
	Govt trng scheme		24		0.15	
	Something else		106		0.68	

### Analysis

A factorial ANOVA was used by taking age, sex, marital status, highest educational qualification, political party supported, and employment status as independent variables and overall satisfaction as the dependent variable. The residuals remained after factoring out age, sex, present legal marital status, highest educational qualification, political party supported, and employment status were kept for further analysis. To test the bottom-up theory, a multiple linear regression was used by taking areas of satisfaction including health, income of household, house/flat, spouse/partner, job, social life, amount of leisure time, and use of leisure time as predictors and the overall satisfaction after factoring out demographics as the predicted variable. To test the top-down hypothesis, we first reversed scores of item optrt5a1 (is sometimes rude to others), optrt5c2 (tends to be lazy), optrt5e3 (is reserved) and optrt5n3 (is relaxed, handles stress well) as these questions were asked in the opposite direction of the corresponding trait. Then we added the sub-items up to get a summary score of each trait. Next, another multiple linear regression was used by taking personality traits including Neuroticism, Openness, Agreeableness, Conscientiousness and Extraversion as predictors and the overall satisfaction as the predicted variable, after factoring out demographics. Finally, we entered demographics, areas of life satisfaction, and personality into a single generalized linear model with life satisfaction as the predicted variable.

## Results

### Demographics and various overall life satisfaction

We found a significant main effect of sex (*F*(1,5,905) = 6.25, *p* < 0.05) and present legal marital status (*F*(1,5,905) = 3.69, *p* = 0.05). However, the main effect of the age, highest educational qualification, political party supported, and employment status were not significant on the overall life satisfaction (Table 2).

**Table 2:** The ANOVA results with the sum of squares, degrees of freedom, mean square, F-stat values, and values of *p* for demographics.

Variables	SumSq	DF	MeanSq	F	*p*-Value
Age	0.96	1	0.96	0.81	0.37
Sex	7.40	1	7.40	6.25	<0.05
Present legal marital status	4.37	1	4.37	3.69	=0.05
Highest educational qualification	1.00	1	1.00	0.84	0.36
Political party supported	1.50	1	1.50	1.27	0.26
Employment status	3.71	1	3.71	3.13	0.08
Error	6997.9	5,905	1.19		

Demographics and overall life satisfaction.

### Areas of satisfaction and overall satisfaction residuals after factoring out demographics

The independent variables explained 51.4% (*R*^2^ = 0.514) of the overall satisfaction score. The variable with the highest impact on the overall satisfaction score was satisfaction with spouse/partner (*β* = 0.18; *t* = 22.17, *p* < 0.001, 95% C.I. [0.17, 0.20]), followed by satisfaction with social life (*β* = 0.13; *t* = 13.30, *p* < 0.001, 95% C.I. [0.11, 0.15]), satisfaction with use of leisure time (*β* = 0.12; *t* = 12.60, *p* < 0.001, 95% C.I. [0.10, 0.14]), satisfaction with health (*β* = 0.11; *t* = 14.93, *p* < 0.001, 95% C.I. [0.09, 0.12]), satisfaction with job (*β* = 0.10; *t* = 14.45, *p* < 0.001, 95% C.I. [0.09, 0.12]), satisfaction with income of household (*β* = 0.07; *t* = 9.19, *p* < 0.001, 95% C.I. [0.05, 0.08]), satisfaction with house/flat (*β* = 0.06; *t* = 7.24, *p* < 0.001, 95% C.I. [0.04, 0.07]), and satisfaction with amount of leisure time (*β* = 0.04; *t* = 4.55, *p* < 0.001, 95% C.I. [0.02, 0.05]; Table 3).

**Table 3:** Multiple regression analysis results for areas of life satisfaction and overall satisfaction after factoring out demographics.

Variables	*β*	SE	*t*-Stat	*p*-Value	95% C.I.
(Intercept)	−4.18	0.06	−71.75	<0.001	[−4.29 –4.06]
Satisfaction with health	0.11	0.01	14.93	<0.001	[0.09, 0.12]
Satisfaction with income of household	0.07	0.01	9.19	<0.001	[0.05, 0.08]
Satisfaction with house/flat	0.06	0.01	7.24	<0.001	[0.04, 0.07]
Satisfaction with spouse/partner	0.18	0.01	22.17	<0.001	[0.17, 0.20]
Satisfaction with job	0.10	0.01	14.45	<0.001	[0.09, 0.12]
Satisfaction with social life	0.13	0.01	13.3	<0.001	[0.11, 0.15]
Satisfaction with amount of leisure time	0.04	0.01	4.55	<0.001	[0.02, 0.05]
Satisfaction with use of leisure time	0.12	0.01	12.60	<0.001	[0.10, 0.14]

### Personality and overall satisfaction residuals after factoring out demographics

The independent variables explained 14.8% (*R*^2^ = 0.148) variances of the overall satisfaction score. The variable with the highest impact on the overall satisfaction score was Neuroticism (*β* = − 0.19; *t* = −19.32, *p* < 0.001, 95% C.I. [−0.21, −0.17]), followed by Conscientiousness (*β* = 0.14; *t* = 10.65, *p* < 0.001, 95% C.I. [0.12, 0.17]), Openness (*β* = 0.15; *t* = 10.56, *p* < 0.001, 95% C.I. [0.12, 0.17]), Agreeableness (*β* = 0.06; *t* = 4.92, *p* < 0.001, 95% C.I. [0.03, 0.08]), and (*β* = 0.04; *t* = 3.30, *p* < 0.001, 95% C.I. [0.02, 0.06]; Table 4).

**Table 4:** Multiple regression analysis results for personality traits and overall satisfaction after factoring out demographics.

Variables	*β*	SE	*t*-Stat	*p*-Value	95% C.I.
(Intercept)	−1.32	0.10	−12.62	< 0.001	[−1.53, −1.12]
Neuroticism	−0.19	0.01	−19.32	< 0.001	[−0.21, −0.17]
Openness	0.15	0.01	10.56	< 0.001	[0.12, 0.17]
Agreeableness	0.06	0.01	4.92	< 0.001	[0.03, 0.08]
Conscientiousness	0.14	0.01	10.65	< 0.001	[0.12, 0.17]
Extraversion	0.04	0.01	3.30	< 0.001	[0.02, 0.06]

### An integrated account of life satisfaction with demographics, areas of life satisfaction, and personality as predictors

Demographics, areas of life satisfaction, and personality traits explained 53% (*R*^2^ = 0.53) variances of total life satisfaction (Table 5). The variable with the highest impact on the overall satisfaction score was satisfaction with spouse/partner (*β* = 0.19; *t* = 21.39, *p* < 0.001, 95% C.I. [0.18, 0.21]), followed by satisfaction with social life (*β* = 0.13; *t* = 12.17, *p* < 0.001, 95% C.I. [0.11, 0.15]), satisfaction with use of leisure time (*β* = 0.13; *t* = 12.06, *p* < 0.001, 95% C.I. [0.10, 0.15]), satisfaction with job (*β* = 0.11; *t* = 13.32, *p* < 0.001, 95% C.I. [0.09, 0.12]), satisfaction with health (*β* = 0.10; *t* = 12.30, *p* < 0.001, 95% C.I. [0.08, 0.11]), satisfaction with income of household (*β* = 0.07; *t* = 9.16, *p* < 0.001, 95% C.I. [0.06, 0.09]), satisfaction with house/flat (*β* = 0.06; *t* = 7.14, *p* < 0.001, 95% C.I. [0.04, 0.08]), satisfaction with amount of leisure time (*β* = 0.04; *t* = 4.88, *p* < 0.001, and 95% C.I. [0.03, 0.06]). Regarding personality traits in this integrative mode, the variable with the highest impact on the overall satisfaction score was Neuroticism (*β* = −0.08; *t* = −9.39, *p* < 0.001, 95% C.I. [−0.10, −0.07]), followed by Openness (*β* = 0.04; *t* = 3.47, *p* < 0.001, 95% C.I. [0.02, 0.07]) and Conscientiousness (*β* = 0.03; *t* = 12.17, *p* < 0.001, 95% C.I. [0.01, 0.06]). However, Agreeableness (*β* = 0.00; *t* = 0.43, *p* = 0.66, 95% C.I. [−0.01, 0.02]) and Extraversion (*β* = 0.01; *t* = 0.75, *p* = 0.45, 95% C.I. [−0.01, 0.03]) were not significant.

**Table 5:** Multiple regression analysis results with overall satisfaction as the predicted variable and demographics, areas of life satisfaction and personality traits as predictors.

Variables	*β*	SE	*t*-Stat	*p*-Value	95% C.I.
(Intercept)	0.93	0.12	7.81	<0.001	[0.70, 1.17]
Age	−0.01	0.00	−5.39	<0.001	[−0.01, −0.00]
Sex	0.08	0.02	3.91	<0.001	[0.04, 0.13]
Present legal marital status	−0.02	0.01	−2.62	<0.01	[−0.03, −0.00]
Highest educational qualification	−0.01	0.00	−1.52	0.13	[−0.01, 0.00]
Political party supported	0.00	0.00	1.32	0.19	[−0.00, 0.01]
Employment status	0.02	0.01	1.82	0.07	[−0.00, 0.03]
Satisfaction with health	0.10	0.01	12.30	<0.001	[0.08, 0.11]
Satisfaction with income of household	0.07	0.01	9.16	<0.001	[0.06, 0.09]
Satisfaction with house/flat	0.06	0.01	7.14	<0.001	[0.04, 0.08]
Satisfaction with spouse/partner	0.19	0.01	21.39	<0.001	[0.18, 0.21]
Satisfaction with job	0.11	0.01	13.32	<0.001	[0.09, 0.12]
Satisfaction with social life	0.13	0.01	12.17	<0.001	[0.11, 0.15]
Satisfaction with amount of leisure time	0.04	0.01	4.88	<0.001	[0.03, 0.06]
Satisfaction with use of leisure time	0.13	0.01	12.06	<0.001	[0.11, 0.15]
Neuroticism	−0.08	0.01	−9.39	<0.001	[−0.10, −0.07]
Openness	0.04	0.01	3.47	<0.001	[0.02, 0.07]
Agreeableness	0.00	0.01	0.43	0.66	[−0.01, 0.02]
Conscientiousness	0.03	0.01	2.86	<0.001	[0.01, 0.06]
Extraversion	0.01	0.01	0.75	0.45	[−0.01, 0.03]

### Correlations between areas of life satisfaction, personality, and overall life satisfaction

Satisfaction with income of household (*r* = 0.42, *p* < 0.001, 95% C.I. [0.40, 0.45]), health (*r* = 0.37, *p* < 0.001, 95% C.I. [0.34, 0.39]), spouse/partner (*r* = 0.24, *p* < 0.001, 95% C.I. [0.22, 0.27]), job (*r* = 0.33, *p* < 0.001, 95% C.I. [0.30, 0.35]), house/flat (*r* = 0.30, *p* < 0.001, 95% C.I. [0.27, 0.33]), amount of leisure time (*r* = 0.66, *p* < 0.001, 95% C.I. [0.64, 0.68]), social life (*r* = 0.59, *p* < 0.001, 95% C.I. [0.57, 0.61]) and use of leisure time (*r* = 0.53, *p* < 0.001, 95% C.I. [0.51, 0.55]) were significantly correlated with the overall satisfaction score. A significant positive correlation was observed between Conscientiousness (*r* = 0.19, *p* < 0.001, 95% C.I. [0.16, 0.22]), Agreeableness (*r* = 0.16, *p* < 0.001, 95% C.I. [0.13, 0.18]), Extraversion (*r* = 0.08, *p* < 0.001, 95% C.I. [0.05, 0.11]), Openness (*r* = 0.16, *p* < 0.001, 95% C.I. [0.13, 0.18]) and overall satisfaction, whereas Neuroticism (*r* = −0.06, *p* < 0.05, 95% C.I. [−0.09, −0.04]) had a significant negative correlation with overall satisfaction. The lowest correlation was found between the Neuroticism and overall life satisfaction (*r* = −0.06, *p* < 0.05, 95% C.I. [−0.09, −0.04]). All correlations can be found in Table 6.

**Table 6:** Correlation between Neuroticism, Openness, Agreeableness, Conscientiousness, Extraversion, satisfaction with health, satisfaction with income of household, satisfaction with house/flat, satisfaction with spouse/partner, satisfaction with job, satisfaction with social life, satisfaction with amount of leisure time, satisfaction with use of leisure time, and overall satisfaction.

Variables	Neuroticism	Openness	Agreeableness	Conscientiousness	Extraversion	Satisfaction with health	Satisfaction with income of household	Satisfaction with house/flat	Satisfaction with spouse/partner	Satisfaction with job	Satisfaction with social life	Satisfaction with amount of leisure time	Satisfaction with use of leisure time
Overall satisfaction	−0.06^*^ [−0.09, −0.04]	0.16^**^ [0.13, 0.18]	0.16^**^ [0.13, 0.18]	0.19^**^ [0.16, 0.22]	0.08^**^ [0.05, 0.11]	0.37^**^ [0.34, 0.39]	0.42^**^ [0.40, 0.45]	0.30^**^ [0.27, 0.33]	0.24^**^ [0.22, 0.27]	0.33^**^ [0.30, 0.35]	0.59^**^ [0.57, 0.61]	0.66^**^ [0.64, 0.68]	0.53^**^ [0.51, 0.55]
Satisfaction with use of leisure time	−0.15^**^ [−0.18, −0.13]	0.39^**^ [0.37, 0.42]	0.29^**^ [0.27, 0.32]	0.20^**^ [0.17, 0.23]	0.07^**^ [0.04, 0.10]	0.28^**^ [0.25, 0.31]	0.18^**^ [0.15, 0.21]	0.32^**^ [0.29, 0.35]	0.33^**^ [0.30, 0.36]	0.31^**^ [0.28, 0.33]	0.66^**^ [0.64, 0.68]	0.46^**^ [0.44, 0.48]	
Satisfaction with amount of leisure time	−0.14^**^ [−0.16, −0.11]	0.19^**^ [0.16, 0.21]	0.08^**^ [0.05, 0.10]	0.15^**^ [0.12, 0.17]	0.06^*^ [0.03, 0.09]	0.18^**^ [0.16, 0.21]	0.39^**^ [0.36, 0.41]	0.33^**^ [0.31, 0.36]	0.22^**^ [0.19, 0.24]	0.29^**^ [0.27, 0.32]	0.55^**^ [0.53, 0.58]		
Satisfaction with social life	−0.07^**^ [−0.01, −0.04]	0.15^**^ [0.12, 0.18]	0.05 [0.03, 0.08]	0.22^**^ [0.20, 0.25]	0.08^**^ [0.05, 0.11]	0.26^**^ [0.23, 0.29]	0.35^**^ [0.32, 0.38]	0.29^**^ [0.26, 0.31]	0.27^**^ [0.24, 0.30]	0.44^**^ [0.41, 0.46]			
Satisfaction with job	−0.25^**^ [−0.27, −0.22]	0.11^**^ [0.08, 0.13]	0.06^*^ [0.04, 0.09]	0.20^**^ [0.18, 0.23]	0.10^**^ [0.07, 0.13]	0.33^**^ [0.30, 0.36]	0.31^**^ [0.29, 0.34]	0.34^**^ [0.31, 0.36]	0.44^**^ [0.42, 0.47]				
Satisfaction with spouse/partner	−0.18^**^ [−0.21, −0.16]	0.17^**^ [0.14, 0.20]	0.07^**^ [0.04, 0.10]	0.23^**^ [0.20, 0.26]	0.14^**^ [0.12, 0.17]	0.26^**^ [0.23, 0.29]	0.33^**^ [0.30, 0.35]	0.41^**^ [0.38, 0.44]					
Satisfaction with house/flat	−0.16^**^ [−0.19, −0.13]	0.20^**^ [0.17, 0.23]	0.10^**^ [0.07, 0.12]	0.19^**^ [0.16, 0.22]	0.06^*^ [0.03, 0.09]	0.32^**^ [0.29, 0.35]	0.43^**^ [0.40, 0.45]						
Satisfaction with income of household	−0.09^**^ [−0.11, −0.06]	0.17^**^ [0.14, 0.20]	0.22^**^ [0.20, 0.25]	0.09^**^ [0.06, 0.12]	0.12^**^ [0.09, 0.15]	0.42^**^ [0.39, 0.45]							
Satisfaction with health	−0.19^**^ [−0.22, −0.17]	0.20^**^ [0.17, 0.22]	0.13^**^ [0.11, 0.16]	0.17^**^ [0.14, 0.20]	0.13^**^ [0.10, 0.16]								
Extraversion	−0.20^**^ [−0.22, −0.17]	0.14^**^ [0.11, 0.17]	0.15^**^ [0.12, 0.18]	0.26^**^ [0.23, 0.28]									
Conscientiousness	−0.18^**^ [−0.20, −0.15]	0.16^**^ [0.13, 0.19]	0.16^**^ [0.13, 0.18]										
Agreeableness	−0.22^**^ [−0.24, −0.19]	0.24^**^ [0.21, 0.26]											
Openness	−0.27^**^ [−0.29, −0.24]												

* *p* < 0.05;

** *p* < 0.001.

## Discussion

The goal of this study was to further research on the relationship between life satisfaction and theories that may affect life satisfaction (bottom-up and top-down) in a large United Kingdom cohort. We found a significant main effect of sex and present legal marital status on overall satisfaction. However, the main effect of the age, highest educational qualification, political party supported, and employment status were not significant on the overall life satisfaction.

Another finding in the present study relates to the association between life satisfaction variables and overall life satisfaction. Our current findings demonstrated that areas of life satisfaction are related to overall life satisfaction (bottom-up theory). Specifically, we found that 51.4% (*R*^2^ = 0.514) of the variances of overall life satisfaction could be explained by life satisfaction variables. In particular, we found that satisfaction with spouse/partner (*β* = 0.18) had the greatest impact on overall satisfaction, followed by satisfaction with social life (*β* = 0.13), satisfaction with use of leisure time (*β* = 0.12), satisfaction with health (*β* = 0.11) and satisfaction with job (*β* = 0.10). Furthermore, satisfaction with income of household (*β* = 0.07), satisfaction with house/flat (*β* = 0.06) and satisfaction with amount of leisure time (*β* = 0.04) contributes to overall satisfaction. In addition, we found that satisfaction with income of household, health, spouse/partner, job, house/flat, amount of leisure time, social life and use of leisure time were significantly correlated with the overall satisfaction score.

Our current findings demonstrated that personality is related to overall life satisfaction, which supports the top-down theory. The personality traits in the regression model explained 14.8% (*R*^2^ = 0.148) of the total life satisfaction variances. Neuroticism (*β* = −0.19), followed by Openness (*β* = 0.15), Conscientiousness (*β* = 0.14), Agreeableness (*β* = 0.06) and Extraversion (*β* = 0.04) contributed to the overall satisfaction score. Moreover, consistent with previous findings in the literature (Diener, 1984; DeNeve and Cooper, 1998; Heller et al., 2004), we showed a significant positive correlation between Agreeableness, Extraversion, Conscientiousness, Openness and overall satisfaction, whereas Neuroticism had a significant negative correlation with overall satisfaction. The lowest correlation was found between the Neuroticism and overall life satisfaction.

Lachmann et al. (2017) provided an explanation regarding the much less variances explained by personality in overall life satisfaction compared to the amount that the areas of life satisfaction could explain. Diener and Diener (2009) and then Diener et al. (2010) emphasized the necessity of investigating not only the direct link between personality and overall life satisfaction, but also interactional and indirect effects (such as the presence or absence of various life circumstances). Additionally, several authors found that individual characteristics have moderating or mediating effects on the relationship between personality and total life satisfaction. For example, Gutiérrez et al. (2005) emphasized the need of taking demographic factors into account. Magee et al. (2013) examined the impact of cultural background on personality and life satisfaction. As a result, it appears that the link between personality and total life happiness is a complicated network containing both direct and indirect paths. If our findings are proven to be consistent with the current literature, future researchers may be motivated to adopt this approach to assess life satisfaction on a wide scale more frequently in life satisfaction research. Moreover, a number of other factors may play a role in the relationship between personality and overall life satisfaction: for example, health situation, level of physical fitness and the presence of diseases. If all of these elements have a role in determining total life happiness, a single factor is likely to contribute only a small portion of the overall score. As a result, the more predictors are used to define a criterion, the more difficult it should be to obtain reliable findings.

Furthermore, we added a model with residuals after factoring out demographics as the predicted variable and areas of life satisfaction, personality traits and demographic variables as predictors. In particular, in this integrative approach the independent variables explained 53.0% (*R*^2^ = 0.530) of the overall satisfaction score. Surprisingly, we found that Agreeableness (*β* = 0.00) and Extraversion (*β* = 0.01) were not significant predictors of the overall life satisfaction in this model. It is certainly possible that certain areas of life satisfaction might moderate the relationship between personality and overall life satisfaction.

The current study has some limitations. First, because we utilized a cross-sectional design, we cannot draw inferences regarding the causality of the relationship between the factors included. Second, data were collected within self-reported measures, which is a tangible risk of biases (Wold et al., 2013). Self-reported measures could be poor indicators of areas of life satisfaction (Rosenman et al., 2011). They may be considered favorable indicators in individualistic societies, where a personal, goal-oriented viewpoint contributes to greater self-referred attribution of failure and success, resulting in higher overall life satisfaction as compared to those in collectivistic cultures (Park et al., 2004; Loewe et al., 2014). However, self-report may still differentiate between different groups of people with regard to broad levels of areas of life satisfaction and personality traits. Third, data is quite old. Therefore, it is possible that the time and the habits present at the time of the collection of the data have influenced the nature of the self-reported measures. Mover, dispositional factors other than personality characteristics are also deemed important for overall life satisfaction. For example, situational elements such as critical life experiences or other environmental effects, have been demonstrated to be relevant in determining one’s degree of life satisfaction. In particular, a recent meta-analysis found that life experiences had an impact on cognitive well-being (Luhmann et al., 2012).

Besides the limitations mentioned above, our findings support an integrative approach to a life satisfaction model (Busseri, 2015; Kuykendall et al., 2015), in which both life satisfaction and personality traits contribute to the overall life satisfaction score. Personality explained 14.8% (*R*^2^ = 0.148) of variances of overall life satisfaction after taking demographic intro account, although larger than the previous study (*R*^2^ = 0.098; Lachmann et al., 2017), it was still substantially lower than the greater 51.4% (*R*^2^ = 0.514) of all life satisfaction variables. Our integrated model with demographic, personality, and areas of life satisfaction as predictors explained 53.0% (*R*^2^ = 0.530) of overall life satisfaction. These findings show that neither a bottom-up nor a top-down perspective alone can adequately explain life satisfaction, as various earlier studies have claimed (Heller et al., 2004; Luhmann et al., 2012; Lachmann et al., 2017). Rather, an integrated account of life satisfaction should be favored.

In conclusion, we found that demographics, personality, and areas of life satisfaction could explain a significant portion of variances in overall life satisfaction. Thus, rather than a unitary bottom-up or top-down model of satisfaction, we propose that an integrated account of life satisfaction should be supported. Future research needs to examine the underlying mechanisms between these associations and also establish causal relationships if possible.

## Data availability statement

Publicly available datasets were analyzed in this study. This data can be found at: https://www.iser.essex.ac.uk/bhps.

## Ethics statement

The studies involving human participants were reviewed and approved by the University of Essex. Written informed consent to participate in this study was provided by the participants’ legal guardian/next of kin.

## Author contributions

AM: writing—original draft and writing—review and editing. WK: conceptualization, data curation, formal analysis, investigation, methodology, project administration, resources, software, supervision, writing—original draft, and writing—review and editing. All authors contributed to the article and approved the submitted version.

## Funding

This work is supported by the Imperial Open Access Fund.

## Conflict of interest

The authors declare that the research was conducted in the absence of any commercial or financial relationships that could be construed as a potential conflict of interest.

## Publisher’s note

All claims expressed in this article are solely those of the authors and do not necessarily represent those of their affiliated organizations, or those of the publisher, the editors and the reviewers. Any product that may be evaluated in this article, or claim that may be made by its manufacturer, is not guaranteed or endorsed by the publisher.
